# Suicidal risks in state of alcoholic drunkenness

**DOI:** 10.1192/j.eurpsy.2022.2180

**Published:** 2022-09-01

**Authors:** L. Baranskaya, Y. Babyshkina

**Affiliations:** 1 Ural State Medical University, Psychiatry, Psychotherapy And Narcology, Yekaterinburg, Russian Federation; 2 Ural State Medical University, Psychiatry, Psychotherapy Fnd Narcology, Yekaterinburg, Russian Federation

## Abstract

**Introduction:**

The last two decades have seen the timeliness of studying the connection between suicides and drunkenness

**Objectives:**

To evaluate the significance of suicidal risk factors in patients who had committed suicides while being under the effect of alcohol so as to be able to forecast suicidal risks and prevent suicides within this group

**Methods:**

The authors have carried out an analysis of medical documentation of suicides committed in the Sverdlovsk region. The data on suicides has been taken from forensic expertise acts. The following factors have been taken into account: age, gender, social status of suicide victim, supplementary somatic pathology, and concentration of alcohol in the victim’s blood

**Results:**

Alcoholic addiction is a behavioral indicator of suicidal risk. The level of suicidal activity in persons with the syndrome of alcoholic addiction is much higher than within the general populace. The age of 25-49 is the peak of suicidal attempts among patients with chronic alcoholism. Genuine suicides prevail during the first stage of chronic alcoholism. The patients are inclined to demonstrate pathological suicidal reactions to social misplacement that show themselves in the form of conflicts within the family and at work. In addition to genuine suicidal attempts made by males in the state of abstinence

**Conclusions:**

The results received confirm the role of the alcoholic factor in the formation of suicidal behavior and have the aim of elaborating new forms and methods to help prevent suicides committed in the state of alcoholic drunkenness

**Disclosure:**

No significant relationships.

